# Compensation for chronic oxidative stress in ALADIN null mice

**DOI:** 10.1242/bio.030742

**Published:** 2018-01-10

**Authors:** Ramona Jühlen, Mirko Peitzsch, Sebastian Gärtner, Dana Landgraf, Graeme Eisenhofer, Angela Huebner, Katrin Koehler

**Affiliations:** 1 Klinik und Poliklinik für Kinder-und Jugendmedizin, Medizinische Fakultät Carl Gustav Carus, Technische Universität Dresden, Dresden 01307, Germany; 2 Institut für Klinische Chemie und Laboratoriumsmedizin, Medizinische Fakultät Carl Gustav Carus, Technische Universität Dresden, Dresden 01307, Germany; 3 Institut für Radiopharmazeutische Krebsforschung, Helmholtz-Zentrum Dresden-Rossendorf, Dresden 01328, Germany; 4 Institut für Klinische Chemie und Laboratoriumsmedizin, Medizinische Klinik und Poliklinik III, Medizinische Fakultät Carl Gustav Carus, Technische Universität Dresden, Dresden 01307, Germany

**Keywords:** ALADIN, Oxidative stress, Paraquat, Redox homeostasis, Triple A syndrome

## Abstract

Mutations in the *AAAS* gene coding for the nuclear pore complex protein ALADIN lead to the autosomal recessive disorder triple A syndrome. Triple A patients present with a characteristic phenotype including alacrima, achalasia and adrenal insufficiency. Patient fibroblasts show increased levels of oxidative stress, and several *in vitro* studies have demonstrated that the nucleoporin ALADIN is involved in both the cellular oxidative stress response and adrenal steroidogenesis. It is known that ALADIN knock-out mice lack a phenotype resembling human triple A syndrome. The objective of this study was to determine whether the application of chronic oxidative stress by ingestion of paraquat would generate a triple A-like phenotype in ALADIN null mice. Adult male mice were fed either a paraquat (0.25 g/kg diet) or control diet for 11 days. After application of chronic oxidative stress, ALADIN knock-out mice presented with an unexpected compensated glutathione metabolism, but lacked a phenotype resembling human triple A syndrome. We did not observe increased levels of oxidative stress and alterations in adrenal steroidogenesis in mice depleted for ALADIN. This study stresses the species-specific role of the nucleoporin ALADIN, which in mice involves a novel compensatory mechanism for regulating the cellular glutathione redox response.

## Abstract (precis)

**Summary:** ALADIN knock-out mice present with an unexpected compensated metabolism of glutathione after application of chronic oxidative stress, whilst lacking a phenotype resembling human triple A syndrome.

## INTRODUCTION

Triple A syndrome (OMIM #231550), a rare autosomal recessive disorder, is caused by homozygous or compound heterozygous mutations in the *AAAS* (achalasia-adrenal insufficiency-alacrima syndrome) gene encoding the nucleoporin ALADIN (alacrima-achalasia-adrenal insufficiency neurologic disorder) (Handschug et al., 2001; Tullio-Pelet et al., 2000). ALADIN is anchored within the nuclear pore complex by the transmembrane nucleoporin NDC1 [nuclear division cycle 1 homologue (*Saccharomyces* *cerevisiae*)] (Kind et al., 2009; Yamazumi et al., 2009). Rabut et al. suggested that ALADIN forms part of the structural backbone of the nuclear pore complex but is not required for the integrity of the complex (Rabut et al., 2004). Triple A patients present with the characteristic triad of adrenocorticotropic hormone-resistant adrenal insufficiency, achalasia of the stomach cardia and alacrima in combination with progressive neurological impairments (Allgrove et al., 1978). Phenotypic appearance of all symptoms is heterogeneous and highly variable. Adrenal atrophy may occur later in life and may develop gradually (Huebner et al., 2002; Milenkovic et al., 2010).

In contrast to other organs with high metabolic rates, the adrenal gland has high levels of enzymatic and non-enzymatic anti-oxidants (Prasad et al., 2014). Imbalances in reactive oxygen species (ROS) result in cellular oxidative stress and have been implicated in a variety of diseases (Prasad et al., 2014). Adrenocortical mitochondrial steroidogenesis increases ROS formation in the cell because the uncoupling of the cytochrome P450 enzyme (CYP) redox reaction can occur at several steps of the reaction (Dekant, 2009; Rapoport et al., 1995). Under these circumstances, superoxide anions and hydrogen peroxide can leak and escape from the redox reaction (Dekant, 2009). Therefore, a balanced level of anti-oxidative mechanisms is crucially important in adrenocortical cells.

It has previously been reported that ALADIN is involved in the cellular oxidative stress response in adrenocortical and fibroblast cells *in vitro*; however, the role of ALADIN in adrenal steroidogenesis and how ALADIN might contribute to adrenal atrophy in triple A patients remains unclear (Hirano et al., 2006; Jühlen et al., 2015; Kind et al., 2010; Koehler et al., 2013; Prasad et al., 2013; Storr et al., 2009). We have shown that depletion of ALADIN in human adrenocortical carcinoma cells leads to an alteration in glucocorticoid and androgen steroidogenesis (Jühlen et al., 2015). Recently, we identified progesterone receptor membrane compartment 2 (PGRMC2) as a novel protein interactor of ALADIN (Jühlen et al., 2016). Microsomal PGRMC2 itself seems to be involved in adrenal steroidogenesis either by regulating heme synthesis, the prosthetic group of microsomal CYPs, or by acting as an electron donor for several CYPs (Piel et al., 2016; Wendler and Wehling, 2013).

Furthermore, in previous work, our group has shown that female homozygous mice deficient for *Aaas* are infertile, while otherwise ALADIN null mice present with a mild phenotype (Huebner et al., 2006). Carvalhal et al. showed that female sterility in ALADIN-deficient mice is caused by impaired chromosomal segregation and maturation of oocytes (Carvalhal et al., 2017). More recently, it was shown that conditional ablation of the ALADIN interactor PGRMC2 from the female reproductive tract results in reproductive senescence (Clark et al., 2016).

Here, we attempted to verify the critical role of ALADIN in the cellular redox regulation using ALADIN null mice. We hypothesized that application of oxidative stress in mice deficient for ALADIN would generate a phenotype similar to that of triple A patients. In order to increase the sensitivity for oxidative stress we used, in addition to our *Aaas* knock-out (KO) mice, offspring from intercrossed heterozygous (Het) *Sod2* (superoxide dismutase 2) and *Aaas* KO mice to obtain *Aaas* KO/*Sod2* Het mice. Because Het SOD2 null mice exhibit increased levels of ROS it seemed reasonable to hypothesize that *Aaas* KO/*Sod2* Het mice would present with increased susceptibility to oxidative stress exposure. Unexpectedly, ALADIN null mice still lacked a phenotype related to triple A syndrome in humans and showed a compensated glutathione metabolism.

## RESULTS

### Chronic oxidative stress is independent of redox-regulated *Hmox1* expression

**Fig. 1.**
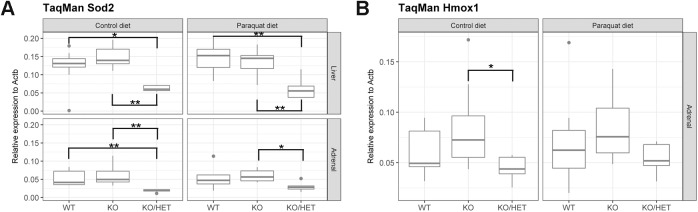
**Expression analysis of redox-regulated adrenal *Hmox1* and adrenal and hepatic *Sod2*.** (A) Adrenal and hepatic *Sod2* and (B) redox-regulated adrenal *Hmox1*. Mice were fed a paraquat diet (0.25 g/kg diet) or a control diet for 11 days. *P<0.05, **P<0.01. Significant differences were measured with unpaired Wilcoxon–Mann–Whitney *U*-test. Boxplot widths are proportional to the square root of the samples sizes. Whiskers indicate the range outside 1.5 times the inter-quartile range above the upper and below the lower quartile. Outliers were plotted as dots.

In order to increase the sensitivity for chronic oxidative stress we used double transgenic *Aaas* KO/*Sod2* Het mice. SOD2 is a mitochondrial antioxidant enzyme essential for transforming and detoxification of free superoxide anions leaking from the mitochondrial aerobic respiration (Li et al., 1995). Mitochondrial SOD2 catalyzes the conversion of free anion radicals to hydrogen peroxide, which in turn can be neutralized by downstream enzymes. Measuring the hepatic and adrenal *Sod2* expression by quantitative real-time polymerase chain reaction (qPCR) we determined, as expected, that *Sod2* expression was about twofold diminished in *Aaas* KO/*Sod2* Het mice under a control or paraquat diet compared to wild-type (WT) and *Aaas* KO mice of the same diet (Fig. 1A).

In the next step, the level of oxidative stress was measured by qPCR of adrenal *Hmox1* gene expression. *Hmox1* is a widely used redox-regulated gene for which transcriptional activation is dependent on upstream transcriptional regulators, which are induced by a broad spectrum of conditions involving oxidative stress, nitrosative stress, thiol-reactive substances and cytokines (Ryter and Choi, 2005). We did not see an increased expression of *Hmox1* in animals under a paraquat diet compared to a control diet (Fig. 1B). However, under the control diet the expression was significantly decreased in *Aaas* KO/*Sod2* Het compared to *Aaas* KO animals.

### Adrenal steroid output is comparable after control and paraquat diet

**Fig. 2.**
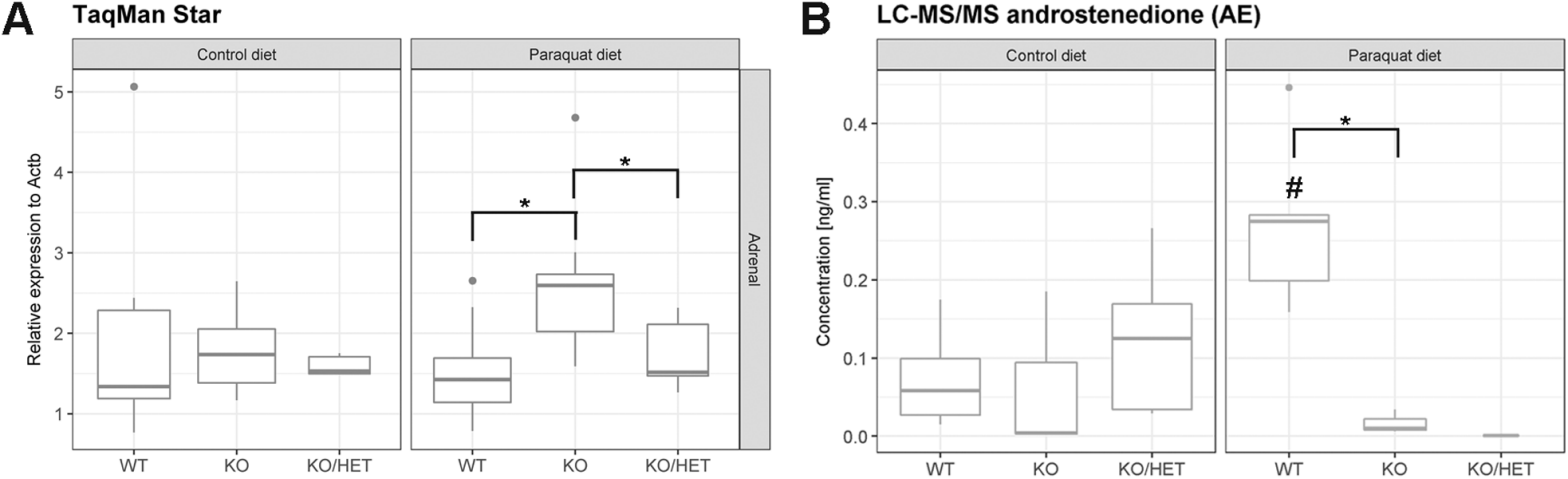
**Oxidative stress affects expression of *Star* and testicular synthesis of androstenedione.** (A) Adrenal expression of *Star* and (B) testicular synthesis of androstenedione. Mice were fed a paraquat diet (0.25 g/kg diet) in the stress group and a control diet in the control group for 11 days. *P<0.05 (between different genotypes in one diet) and ^#^*P*<0.05 (between different diets in one genotype). Significant differences were measured with unpaired Wilcoxon–Mann–Whitney *U*-test. Boxplot widths are proportional to the square root of the samples sizes. Whiskers indicate the range outside 1.5 times the inter-quartile range above the upper and below the lower quartile. Outliers were plotted as dots.

The investigation of the expression of different enzymes of steroidogenesis revealed that the expression of *Star* was increased in *Aaas* KO versus WT animals after paraquat diet (Fig. 2A). Furthermore, *Aaas* KO/*Sod2* Het mice under paraquat diet presented with decreased expression of *Star* compared to *Aaas* KO mice on the same diet. However, neither expression levels of *Cyp21a1*, *Cyp11a1*, *Cyp11b1*, *Cyp11b2* and *Hsd3b2* were changed nor could we see a specific effect depending on the genotype of the mice (Fig. S1A-E).

Plasma levels of pregnenolone, progesterone, deoxycorticosterone, corticosterone, aldosterone and dehydroepiandrosterone sulfate were not significantly altered by paraquat diet or among the different genotypes (Fig. S2A-F). Plasma levels of 17-hydroxyprogesterone and dehydroepiandrosterone were under the detection threshold. Production of androstenedione, which mice only synthesize in the gonads, increased about fivefold in WT animals after ingestion of paraquat compared to WT animals on the control diet (Fig. 2B). Furthermore, androstenedione levels in *Aaas* KO animals decreased about 25-fold after paraquat diet compared with WT animals also assigned a paraquat diet (Fig. 2B).

### Paraquat diet and ALADIN depletion decrease body weight gain

**Fig. 3.**
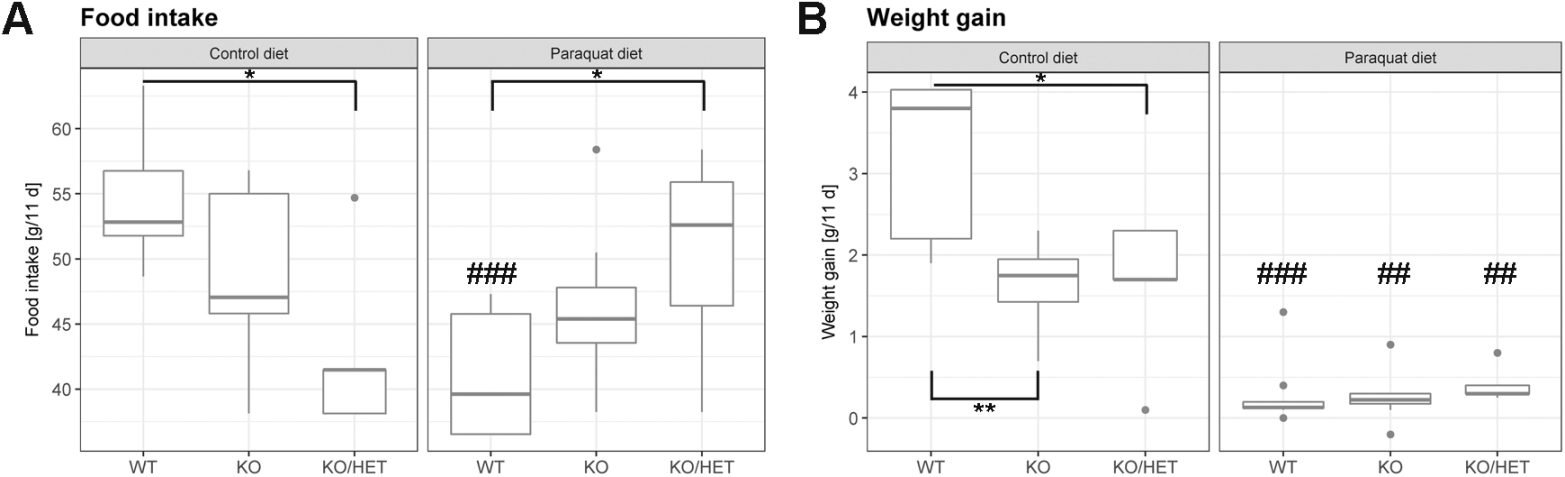
**Alteration of food intake and body weight gain by oxidative stress.** (A) Murine food intake and (B) body weight gain after oxidative stress exposure. Mice were fed a paraquat diet (0.25 g/kg diet) in the stress group and a control diet in the control group for 11 days. Body and diet weight were determined every day during the feeding period. *P<0.05, **P<0.01 (between different genotypes in one diet) and ^##^*P*<0.01, ^###^*P*<0.001 (between different diets in one genotype). Significant differences were measured with unpaired Wilcoxon–Mann–Whitney *U*-test. Boxplot widths are proportional to the square root of the samples sizes. Whiskers indicate the range outside 1.5 times the inter-quartile range above the upper and below the lower quartile. Outliers were plotted as dots.

In the mice fed the control diet, food intake over an 11-day period significantly decreased in *Aaas* KO/*Sod2* Het mice compared to WT mice (Fig. 3A). There was also a slight decline in intake in *Aaas* KO animals. Weight gain in *Aaas* KO and *Aaas* KO/*Sod2* Het mice was about twofold diminished in the control diet compared to the WT animals (Fig. 3B).

WT mice on the paraquat diet consumed less compared to those on the control diet; however, food intake in *Aaas* KO/*Sod2* Het mice was higher versus the WT (Fig. 3A). Accordingly, WT animals gained about 20-fold less weight compared to the control diet and weight gain was also about fourfold lowered in *Aaas* KO and *Aaas* KO/*Sod2* Het animals versus the control diet, despite increased food intake (Fig. 3A,B).

### Hepatic glutathione levels are balanced in ALADIN null mice

**Fig. 4.**
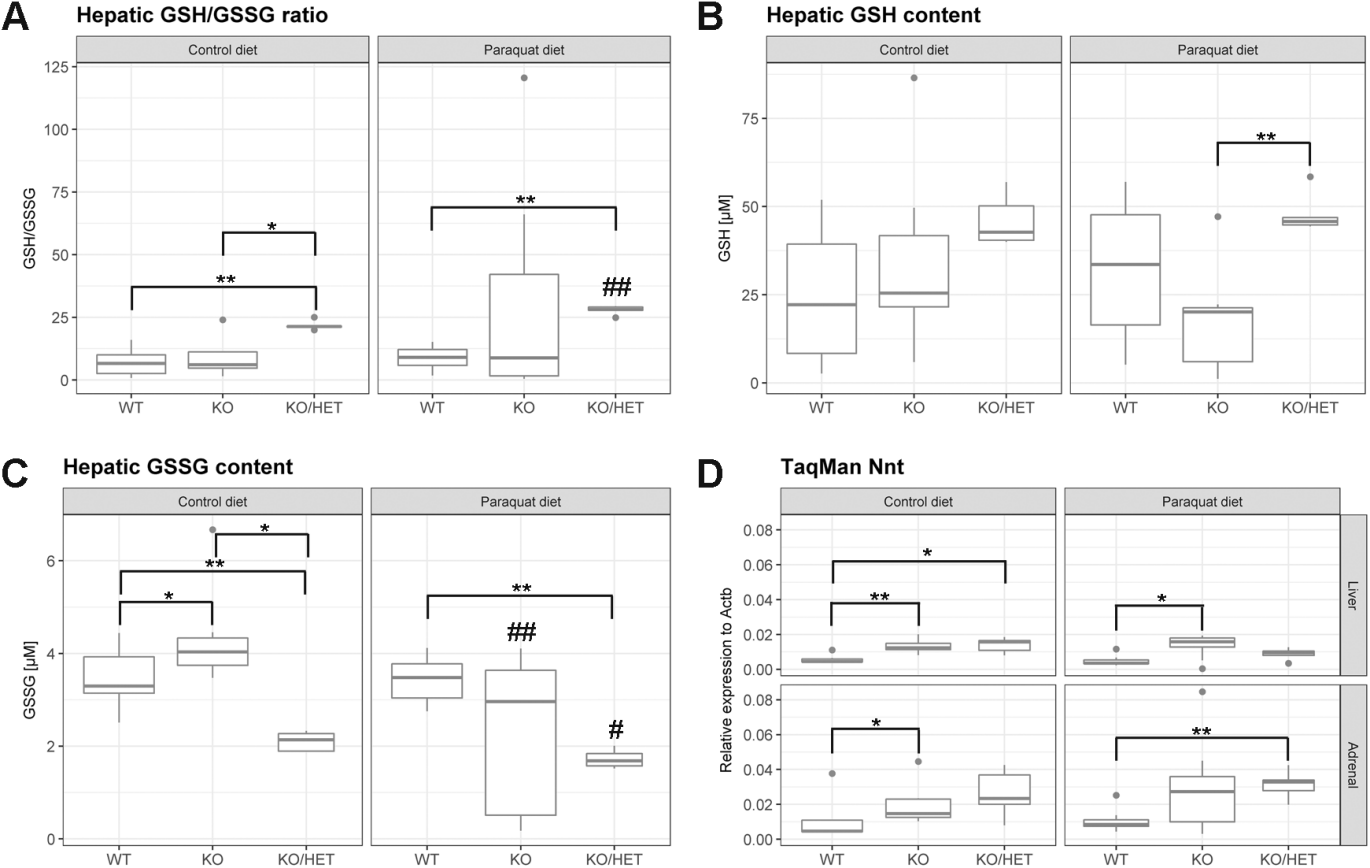
**Balance of hepatic glutathione levels in ALADIN null mice.** Mice were fed a paraquat diet (0.25 g/kg diet) in the stress group and a control diet in the control group for 11 days. GSH, reduced glutathione. GSSG, oxidized glutathione. *P<0.05, **P<0.01 (between different genotypes in one diet) and ^#^*P*<0.05, ^##^*P*<0.01 (between different diets in one genotype). Significant differences were measured with unpaired Wilcoxon–Mann–Whitney *U*-test. Boxplot widths are proportional to the square root of the samples sizes. Whiskers indicate the range outside 1.5 times the inter-quartile range above the upper and below the lower quartile. Outliers were plotted as dots.

Glutathione (GSH) is an important antioxidant in cells and is converted to its oxidized form, glutathione disulfide (GSSG), to detoxify ROS. The ratio of reduced to oxidized glutathione (GSH/GSSG) decreases when cells are exposed to increased levels of oxidative stress; GSSG accumulates and GSH is consumed. Surprisingly, hepatic GSH/GSSG ratios in *Aaas* KO/*Sod2* Het animals on both the control and paraquat diets increased about fivefold compared to WT animals on the same diet (Fig. 4A). Additionally, the paraquat diet increased the ratio significantly in *Aaas* KO/*Sod2* Het animals compared to the same genotype under the control diet. GSH/GSSG ratios of *Aaas* KO mice were similar to those of WT mice.

GSH concentrations were higher in *Aaas* KO/*Sod2* Het livers under either the control or paraquat diet versus WT and *Aaas* KO mice on the same diet (Fig. 4B). Hepatic GSH content in *Aaas* KO mice was comparable to that in WT mice.

Similarly, hepatic GSSG concentrations were about twofold diminished in *Aaas* KO/*Sod2* Het animals on the control diet compared to WT and *Aaas* KO animals on the same diet (Fig. 4C). Furthermore, GSSG concentrations decreased in *Aaas* KO/*Sod2* Het mice on the paraquat diet compared to those in the same animals on the control diet. Interestingly, GSSG content in *Aaas* KO mice on the control diet was significantly higher compared to that in WT and *Aaas* KO/*Sod2* Het mice on the same diet. This effect was reversed under the paraquat diet: GSSG concentration in *Aaas* KO animals decreased compared to that in the same animals on the control diet.

Glutathione peroxidase (GPX) catalyzes the detoxification of hydrogen peroxide to water by producing GSSG. Glutathione reductase (GSR) maintains GSH levels from GSSG by consuming nicotinamide adenine dinucleotide phosphate (NADPH), which in turn is made from nicotinamide adenine dinucleotide (NADH) by mitochondrial nicotinamide nucleotide transhydrogenase (NNT). To verify our previous measurements of hepatic GSH/GSSG ratios, we assessed the hepatic expression of *Gpx1* and *Gsr* as well as the adrenal and hepatic expression of *Nnt*. We could see no alteration in the expression of *Gpx1* in control and paraquat diet animals and in the different genotypes (Fig. S3A). However, *Aaas* KO/*Sod2* Het mice under a paraquat diet presented with decreased expression of *Gsr* compared to *Aaas* KO mice under the same diet (Fig. S3B). Most strikingly, hepatic and adrenal expression of *Nnt* was about twofold increased in *Aaas* KO and *Aaas* KO/*Sod2* Het mice under a control diet versus the WT (Fig. 4D). Under a paraquat diet, hepatic *Nnt* expression was still higher in *Aaas* KO animals compared to WT animals, and adrenal *Nnt* expression was significantly increased in *Aaas* KO/*Sod2* Het mice compared to WT animals under a paraquat diet.

We additionally measured the end-products of hepatic lipid peroxidation (thiobarbituric acid reactive substances, TBARS) to estimate the level of oxidative stress in our animals. Relative liver and lung weights and hepatic TBARS values were not altered upon oxidative stress exposure using a paraquat diet or in the different genotypes (Figs S4 and S5). No pathological differences in histology sections of brain, duodenum, liver and lung could be found (data not shown).

## DISCUSSION

In the present study we investigated the role of the nucleoporin ALADIN in chronic paraquat-induced oxidative stress in male mice. ALADIN-deficient mice lack a triple A syndrome-characteristic phenotype (Huebner et al., 2006). Previous studies have demonstrated that ALADIN employs a crucial role in the redox response of the cell *in vitro* (Hirano et al., 2006; Jühlen et al., 2015; Kind et al., 2010; Koehler et al., 2013; Prasad et al., 2013; Storr et al., 2009). Triple A patients also suffer from increased cellular oxidative stress, as shown by Fragoso et al. (2017). Thus, we hypothesized that chronic oxidative stress would unmask the distinct phenotype in ALADIN null mice.

Overall, we did not see a triple A syndrome-characteristic phenotype in mice depleted for ALADIN after chronic oxidative stress exposure. Prior to this study we performed a pilot experiment inducing acute oxidative stress in mice by injection with paraquat intraperitoneally (25 mg/kg body weight), but no involvement of ALADIN in the acute oxidative stress response was ascertained (data not shown). The results for our *in vivo* murine model do not follow those expected from various human *in vitro* cell systems in which depletion of ALADIN leads to disturbed redox homeostasis and altered adrenal steroidogenesis (Jühlen et al., 2015; Prasad et al., 2013). We assume that this discrepancy is either a result of a possible species-specific role of ALADIN or of the experimental nature of the *in vitro* versus *in vivo* models. The *AAAS* gene is highly conserved between human, mouse (93.6%) and rat (92.3%). The pairwise alignment of *Mus* *musculus* and *Rattus* *norvegicus* *AAAS* sequence revealed a 97.1% homology between these rodents.

**Fig. 5.**
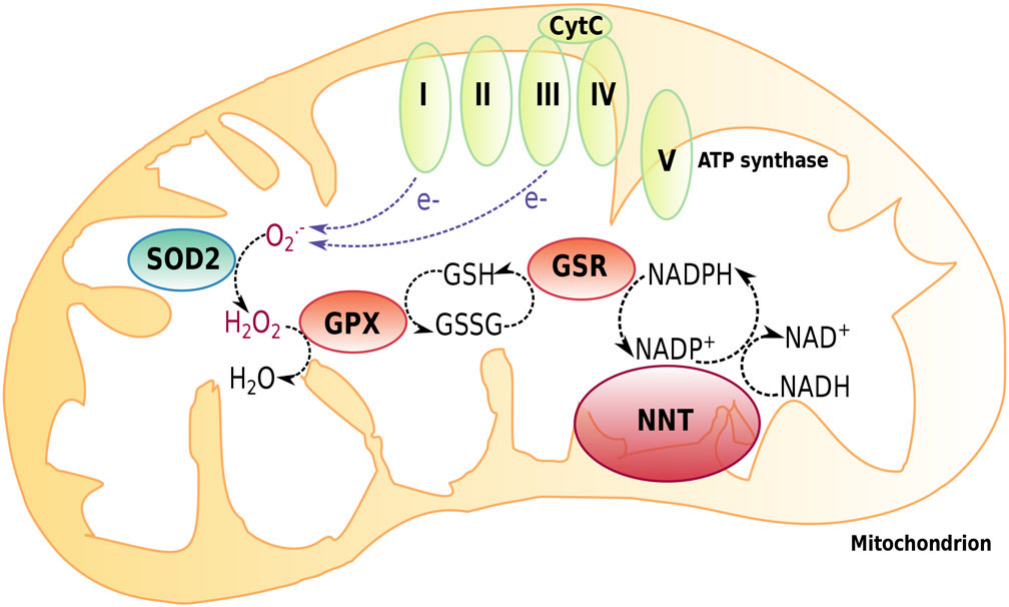
**Mitochondrial redox defense system.** Transmembrane nicotinamide nucleotide transhydrogenase (NNT) contributes to the mitochondrial redox defense system by producing NADPH. NADPH is consumed by glutathione reductase (GSR), maintaining reduced glutathione (GSH) levels from oxidized glutathione (GSSG). Electrons leaking during mitochondrial aerobic respiration result in superoxide anion radicals (O_2_^−^) and are converted to hydrogen peroxide (H_2_O_2_) by mitochondrial superoxide dismutase (SOD2). Hydrogen peroxide is neutralized to water (H_2_O), consuming GSH by glutathione peroxidase (GPX).

Our data indicate that on one hand, mice depleted for ALADIN during basal conditions and after chronic oxidative stress exposure sustain balanced hepatic glutathione levels by upregulation of *Nnt* resulting in a WT-like phenotype. On the other hand, *Aaas* KO/*Sod2* Het mice under basal conditions increase hepatic glutathione levels by increasing *Nnt* expression. This effect was intensified after chronic oxidative stress exposure. In the cell, transmembrane NNT plays a key role in the mitochondrial defense system against ROS by producing NADPH (Fig. 5). NADPH is in turn consumed by GSR), maintaining reduced glutathione (GSH) levels from oxidized glutathione (GSSG) (Krengel and Törnroth-Horsefield, 2015). ROS and in particular superoxide anions leaking during mitochondrial aerobic respiration or produced by exogenous stressors are converted to hydrogen peroxide by mitochondrial superoxide dismutase (SOD2). Hydrogen peroxide is then neutralized to water by consuming GSH by several peroxidases (GPX).

It has already been shown that heterozygous deficiency for *Sod2* in mice activates mitochondrial uncoupling to reduce ROS production and increases aerobic glycolysis by a free radical-mediated mechanism (Xu et al., 2015). Mice heterozygously deficient for *Sod2* exhibit increased levels of ROS and shift from mitochondrial oxidative phosphorylation to a cytosolic glycolytic pathway (Xu et al., 2015). During aerobic glycolysis, a high rate of energy is produced by metabolizing glucose into pyruvate which then feeds into cytosolic lactic acid fermentation rather than mitochondrial oxidation, commonly known as the Warburg effect (Vander Heiden et al., 2009; Xu et al., 2015). It can thus be assumed that the phenotypic effects observed in *Aaas* KO/*Sod2* Het mice in the present study are caused by both the Warburg effect and increased expression of *Nnt*. These additive actions lead to transient increase of glutathione oxidative capacity and to an enhancement of the compensatory effect seen in *Aaas* KO mice, which lack typical symptoms of triple A syndrome. Thus, we suggest that ALADIN plays a crucial role in regulating NADPH levels in the cell and concomitantly enhances oxidative capacity of glutathione by altered gene expression of NNT. Interestingly, it has been demonstrated that both under- or overexpression of *Nnt* reduced corticosterone output in mice, implying a central role for it in the control of steroidogenesis (Meimaridou et al., 2017). Furthermore, gene downregulation of *Nnt* has been associated with age-related neurodegeneration in Alzheimer disease-like mouse neurons (Ghosh et al., 2014). It has been reported that NAD(P)H redox control is more critical than GSH content in promoting neurodegeneration (Ghosh et al., 2014). This result partly explains why mice depleted for ALADIN do not present with a triple A syndrome-distinct phenotype but rather behave like WT animals.

We based our study of chronic paraquat-induced oxidative stress on the work of Aoki and colleagues in which a 0.025% paraquat-enriched diet was also used to induce oxidative stress in 4-week-old juvenile male rats (Aoki et al., 2002). In contrast to our results, Aoki et al. found that rats fed the paraquat diet suffered from elevated hepatic lipid (TBARS) and glutathione (GSSG) oxidation, liver organ shrinkage and lung enlargement (Aoki et al., 2002). We could not reproduce these results in our mice. This may be due to the different age of the animals or to different anti-oxidant defenses in the two rodent species. Results from Aoki et al. regarding food intake and body weight gain were consistent with our study (Aoki et al., 2002). Here, we show that depletion of ALADIN in mice negatively affected body weight gain under normal control and paraquat diet. This result is underlined by increased food intake under paraquat diet in these animals.

Our *in vivo* study is the first to highlight a species-specific role of the nucleoporin ALADIN. The data suggest involvement of a complex cellular system that compensates for the depletion of ALADIN, which seems to have an important role in balancing cellular NADPH levels. Future research on oxidative stress pathways in mice shall address how this possible compensating mechanism functions and may further clarify the role of ALADIN in the pathogenesis of triple A syndrome.

## MATERIALS AND METHODS

### Experimental animals and treatments

All mice were housed in the animal care facility (Experimental Center) of the Technical University Dresden, Dresden, Germany. All procedures were approved by the Regional Board for Veterinarian Affairs, Dresden, Germany (AZ 24-9168.11-1/-2010-49) in accordance with the institutional guidelines for the care and use of laboratory animals. Animals were group housed except during actual experimental procedures, when single housing was required. Mice were kept under specific-pathogen-free conditions at a constant temperature (22±1°C) and a 12 h light-dark cycle at all times. Mice were weaned onto ssniff R/M-H (19% protein, 4.9% fibers, 3.3% fat, 12.2 MJ/kg) (ssniff GmbH, Soest, Germany) if not stated otherwise and drank water *ad libitum*. *Aaas-*deficient mice were generated as described previously (Huebner et al., 2006) and backcrossed to strain C75BL/6J for 10 generations. A heterozygous *Sod2* mouse strain was obtained from The Jackson Laboratory, Bar Harbor, ME USA (Strain #002973 B6.129S7-Sod2^tm1Leb^/J). Heterozygous *Sod2* female mice were intercrossed for two generations with *Aaas* KO male mice to obtain *Aaas* KO/*Sod2* Het mice.

For chronic oxidative stress exposure 1-year-old adult male mice of three different genotypes [wild-type (WT) (*n*=16), *Aaas* KO (*n*=16) and *Aaas* KO/*Sod2* Het (*n*=10)] were used and randomly divided into two groups (stress and control group). All were placed on a commercial diet (ssniff R/M-H) for 3 days to allow acclimation to these conditions. Mice were then fed a paraquat diet (0.25 g/kg diet) (ssniff GmbH) in the stress group and a control diet in the control group (ssniff GmbH) for 11 days. Body weight and diet weight were determined every day during the feeding period. At the end (day 11) of the feeding period animals were sacrificed. Lungs and liver were surgically removed, washed in ice-cold PBS and weighed. Different parts of the liver were prepared for glutathione measurement and assessment of lipid peroxidation. Adrenals and liver sections were surgically excised and quickly frozen in liquid nitrogen and stored at −80°C before RNA extraction.

### Hepatic glutathione assay

Small samples (40-100 mg) of liver tissue were rapidly cut on an ice-cold petri dish to prevent oxidation of GSH to GSSG during preparation. Each small sample was immediately placed with a forceps in liquid nitrogen. Samples in the tubes were re-weighed and the weight of the tissue was determined. Ten volumes of ice-cold 5% sulfosalicylic acid (Carl Roth, Karlsruhe, Germany) were added to each tube, the sample was transferred to a tissue grinder and homogenized until evenly suspended. The suspension was added to the same tube and centrifuged at 4°C at 14,000×***g*** for 10 min. The supernatant was transferred to a new tube and equal volume of ice-cold 500 mM HEPES (pH 8) (Gibco, Thermo Fisher Scientific, Schwerte, Germany) were added.

Each sample was diluted 60-fold in ice-cold 250 mM HEPES (pH 7.5) to be in linear detection range for measurement of total and oxidized glutathione using the GSH/GSSG-Glo assay (Promega, Mannheim, Germany). Measurements were performed in duplicate as outlined in the protocol of the manufacturer and as reported elsewhere (Jühlen et al., 2015).

### Hepatic lipid peroxidation measurement

End-products of hepatic lipid peroxidation, malondialdehyde precursors and other thiobarbituric acid reactive substances (TBARS), were extracted from liver sections as described before by centrifugation at 1600×***g*** for 10 min (Mihara and Uchiyama, 1978). TBARS were quantified in triplicate spectrophotometrically at 535 and 520 nm as outlined previously (Mihara and Uchiyama, 1978) on a 96-well culture dish (200 µl/well) (Corning Costar, Kaiserslautern, Germany) using an Infinite 200 PRO Microplate Reader with the Magellan Data Analysis Software v6.6 (Tecan Group AG, Männedorf, Switzerland).

### RNA extraction, cDNA synthesis and quantitative real-time PCR using TaqMan

Total RNA from frozen murine liver and adrenals was isolated, purity assessed, reverse transcribed and qPCR amplified in 20 μl total volumes as outlined elsewhere (Jühlen et al., 2016). As a reference gene for normalization beta-actin was evaluated and used. Positive controls contained a random mix of cDNA and negative controls contained nuclease-free water instead of cDNA. In all real-time qPCR experiments, relative gene expression was calculated by the C_t_ method using standard and semi-log plots of amplification curves. In all results repeatability was assessed by standard deviation of triplicate C_t_ s and reproducibility was verified by normalizing all real-time reverse transcription (RT)-PCR experiments by the C_t_ of each positive control per run.

Primers for the amplification of the target sequence of beta actin (*Actb*), cytochrome P450 enzyme 11a1 (*Cyp11a1*), *Cyp11b1*, *Cyp11b2*, *Cyp21a1*, glutathione peroxidase 1 (*Gpx*1), glutathione reductase (*Gsr*), heme oxygenase 1 (*Hmox1*), hydroxy-delta-5-steroid dehydrogenase (*Hsd3b2*), nicotinamide nucleotide transhydrogenase (*Nnt*), superoxide dismutase 2 (*Sod2*) and steroidogenic acute regulatory protein (*Star*) were designed using Primer Express 3.0 (Applied Biosystems, Life Technologies, Darmstadt, Germany) and compared to the murine genome database for unique binding using BLAST search (https://blast.ncbi.nlm.nih.gov/Blast.cgi). The primer sequences and gene accession numbers are listed in Table S1.

The guidelines of the Minimum Information for Publication of Quantitative Real-Time PCR Experiments were followed in this study to allow more reliable interpretation of real-time RT-PCR results (Bustin et al., 2013).

### LC-MS/MS measurement of steroids

Blood for plasma steroid measurement by liquid chromatography tandem mass spectrometry (LC/MS-MS) was collected by cardiac puncture. Plasma steroids pregnenolone (Preg), progesterone (P), 17-hydroxyprogesterone (17OHP), deoxycorticosterone (DOC), corticosterone (B), aldosterone (ALDO), androstenedione (AE), dehydroepiandrosterone (DHEA) and dehydroepiandrosterone sulfate (DHEAS) were determined simultaneously by LC-MS/MS as reported previously (Peitzsch et al., 2015). Quantification of steroid levels was performed by comparisons of ratios of analyte peak area obtained from plasma samples to the respective peak area of stable isotope labelled internal standard calibrators.

### Histology

Sections of brain, duodenum, liver and lung were washed in PBS and fixed in 4% formaldehyde (SAV LP, Flinsbach, Germany) for 24 h. Organs were then transferred to PBS and prepared for histology at the Histology Facility of the Joint Technology Platform (Technische Universität Dresden, Biotec, CRTD).

Tissues were embedded into paraffin with the Microm STP 420 D dehydration/infiltration unit (Thermo Fisher Scientific, Waltham, Massachusetts, USA) and the EGF 1160 embedding station (Leica, Wetzlar, Germany). This included stepwise dehydration in a graded alcohol series, transfer to xylol, as well as paraffin infiltration and sample orientation. Paraffin-embedded samples were sectioned using a Microm HM 340E (Thermo Fisher Scientific) and stained with Hematoxylin-Eosin (Carl Roth).

### Statistical analysis

Statistical analyses were conducted using the open-source software R version 3.3.2 and R Studio version 1.0.136 (R Core Team, 2016). Unpaired two-tailed Wilcoxon–Mann–Whitney *U*-test was performed. During evaluation of the results a confidence interval alpha of 95% and *P*-values lower than 0.05 were considered as statistically significant.

## Supplementary Material

Supplementary information
