# Supplementary material for: Compensation for chronic oxidative stress in ALADIN null mice
Source: Biol Open. 2018 Jan 10;7(1):bio030742. doi: 10.1242/bio.030742 (PMC5829508; doi:10.1242/bio.030742)
Supplement: Supplementary information [file biolopen-7-030742-s1.pdf]

Table 1 – Real-time qPCR primer oligonucleotides.

GENE	NAME	SEQUENCE
Actb	m*Actb*-F	GCCAACCGTGAAAAGATGAC
(V1: NM_007393.5)	m*Actb*-R	CATACAGGGACAGCACAGCC
	m*Actb*-Probe	TTTGAGACCTTCAACACCCCAGCCATGT
Cyp11a1	m*Cyp11a1*-F	GAACATCCAGGCCAACATTACC
(V1: NM_019779.4)	m*Cyp11a1*-R	GTTGTGTGCCATCTCATAAAGGTT
	m*Cyp11a1*-Probe	ACACGACCTCCATGACCCTGCAGTG
Cyp11b1	m*Cyp11b1*-F	TAGAGAACTCCGTGGCCTGG
(V1: NM_001033229.3)	m*Cyp11b1*-R	TCAGCTGCAGTCGGTTGAAG
	m*Cyp11b1*-Probe	TCTGGCCCATTTAGCAAGAACACACCA
Cyp11b2	m*Cyp11b2*-F	TCAGGGATCTTTGCCTCTCG
(V1: NM_009991.3)	m*Cyp11b2*-R	TTGCTGTCGTGTCAACGCTC
	m*Cyp11b2*-Probe	AAAGCGAACTCTATGGAGCTCACCGCTG
Cyp21a1	m*Cyp21a1*-F	GGCTGTGGCTTTCCTGCTT
(V1: NM_009995.2)	m*Cyp21a1*-R	CCAGCTTGAGGTCTAACTCTTCCT
	m*Cyp21a1*-Probe	ACCACCCTGAGATCCAGAAGCGACTG
Gpx1	m*Gpx1*-F	CTCGGTTTCCCGTGCAAT
(V1: NM_008160.6)	m*Gpx1*-R	GACGTACTTGAGGGAATTCAGAATC
	m*Gpx1*-Probe	CATTCTTGCCATTCTCCTGGTGTCCG
Gsr	m*Gsr*-F	ACTTGCGTGAATGTTGGATGTG
(V1: NM_010344.4)	m*Gsr*-R	GCCGTAATCCACGTGATCGT
	m*Gsr*-Probe	AATTCCGAGTGCACTGCTGTGTTCCAC
Hmox1	m*Hmox1*-F	CGTGCTCGAATGAACACTCT
(V1: NM_010442.2)	m*Hmox1*-R	GCTCCTCAAACAGCTCAATG
	m*Hmox1*-Probe	CACCCTGTGCTTGACCTCAGGTG
Hsd3b2	m*Hsd3b2*-F	CCCAGGCAGACCATCCTAGA
(V1: NM_153193.3)	m*Hsd3b2*-R	AAGGCTGGCACACTGGCTT
	m*Hsd3b2*-Probe	CAGGCCTCCAATAAGTTCTGGGTACCTTTCAG
Nnt	m*Nnt*-F	ATGCCGGTCCTTGAGGTC
(V2: NM_001308506.1)	m*Nnt*-R	GGTTTGTAGAAGATCGGATTGTC
	m*Nnt*-Probe	AGCCAACGCCAAGGGACCTCTTCATG
Sod2	m*Sod2*-F	AACGCCACCGAGGAGAAGTA
(V1: NM_013671.3)	m*Sod2*-R	CTGAAGAGCGACCTGAGTTGTAAC
	m*Sod2*-Probe	TCTCCCTTGGCCAGAGCCTCGTG
Star	m*Star*-F	CTCACTTGGCTGCTCAGTATTGA
(V1: NM_011485.5)	m*Star*-R	GGTGGTTGGCGAACTCTATCTG
	m*Star*-Probe	AAGACAATCATCAACCAGGTCCTATCGCAG

Biology Open • Supplementary information

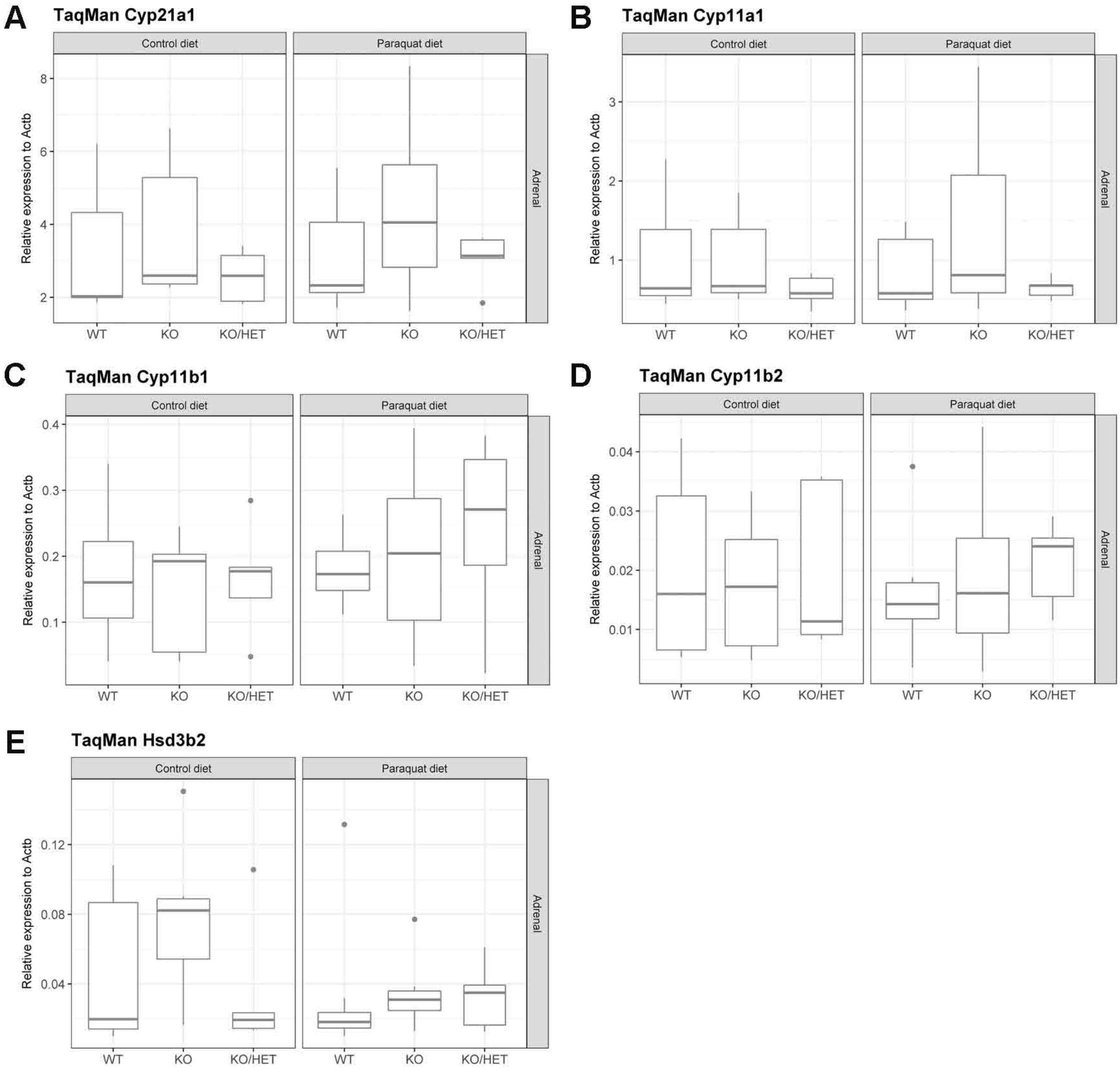

Fig. S1 Adrenal steroidogenesis gene expression after paraquat exposure.

Mice were fed with paraquat diet (0.25 g/kg diet) and with control diet for 11 days. Boxplot widths are proportional to the square root of the samples sizes. Whiskers indicate the range outside 1.5 times the inter-quartile range above the upper and below the lower quartile. Outliers were plotted as dots.

Biology Open • Supplementary information

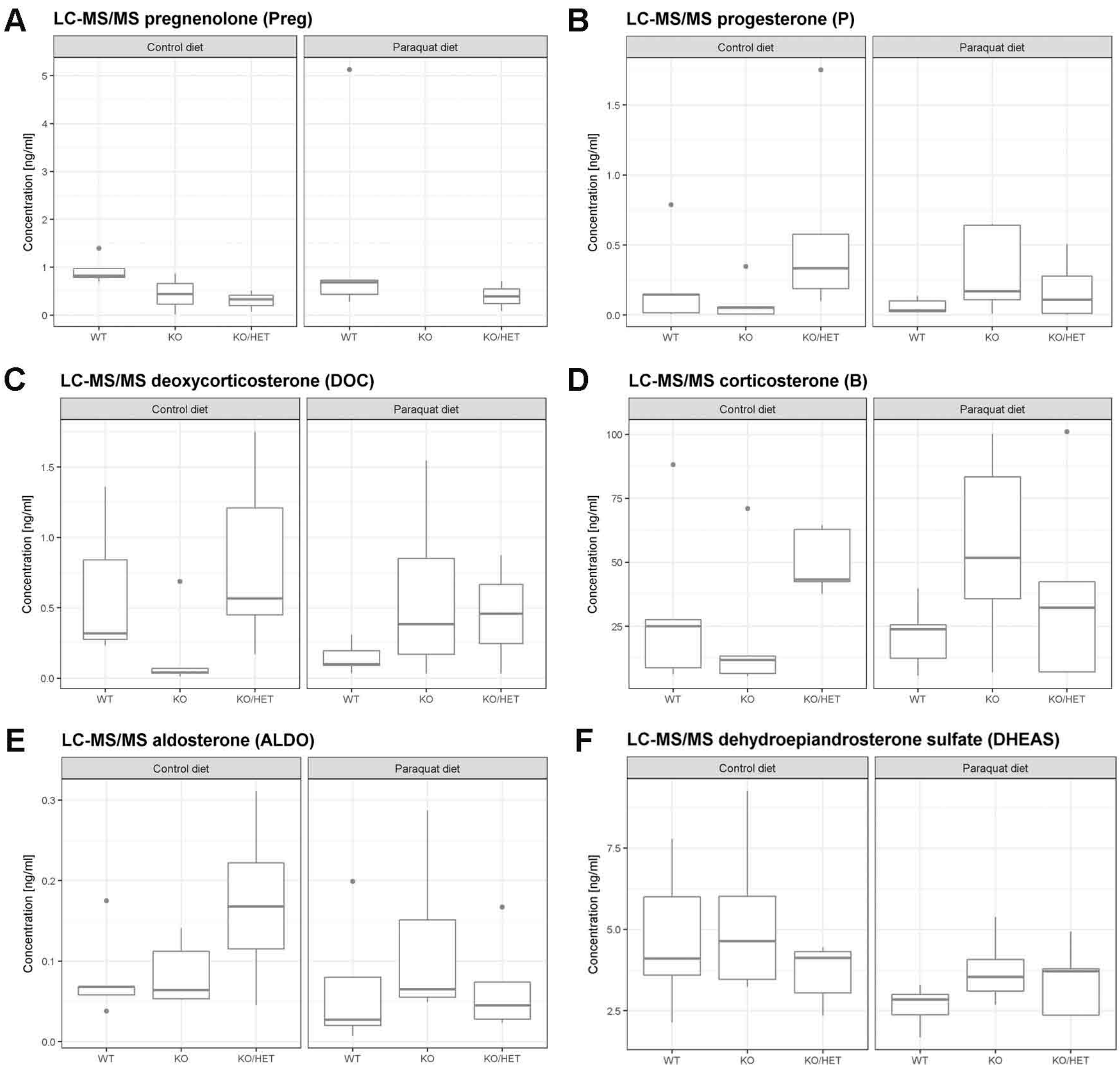

Fig. S2 Steroid output after paraquat exposure.

Mice were fed with paraquat diet (0.25 g/kg diet) and with control diet for 11 days. Boxplot widths are proportional to the square root of the samples sizes. Whiskers indicate the range outside 1.5 times the inter-quartile range above the upper and below the lower quartile. Outliers were plotted as dots.

Biology Open • Supplementary information

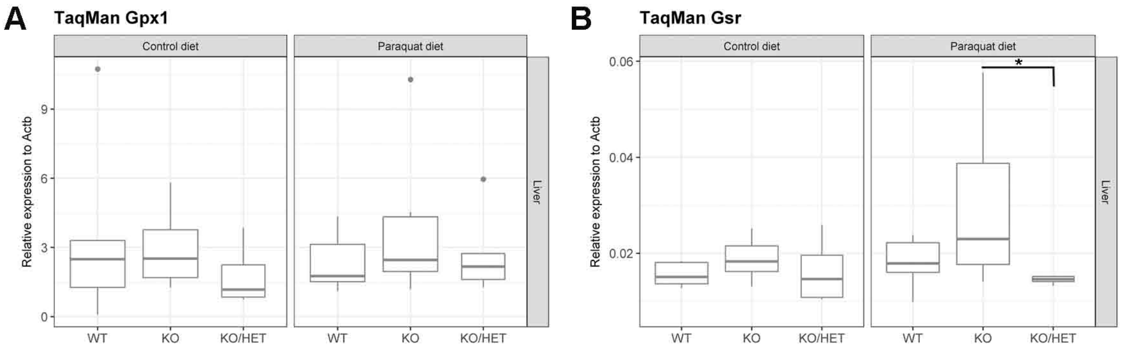

Fig. S3 Expression analysis of hepatic *Gpx1* and *Gsr*.

Mice were fed with paraquat diet (0.25 g/kg diet) and with control diet for 11 days. P-values: * P<0.05. Significant differences were measured with unpaired Wilcoxon–Mann–Whitney U-test. Boxplot widths are proportional to the square root of the samples sizes. Whiskers indicate the range outside 1.5 times the inter-quartile range above the upper and below the lower quartile. Outliers were plotted as dots.

Biology Open • Supplementary information

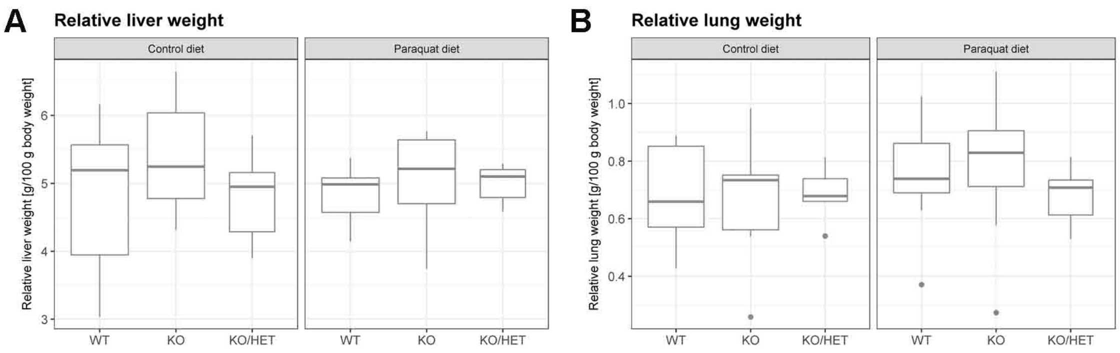

Fig. S4 Relative liver and lung weights are unchanged upon fed diets containing paraquat. Mice were fed with paraquat diet (0.25 g/kg diet) and with control diet for 11 days. Boxplot widths are proportional to the square root of the samples sizes. Whiskers indicate the range outside 1.5 times the inter-quartile range above the upper and below the lower quartile. Outliers were plotted as dots.

Biology Open • Supplementary information

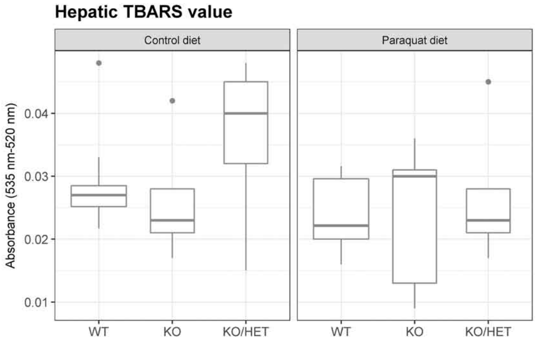

Fig. S5 The level of hepatic lipid peroxidation is not altered after the paraquat diet. Mice were fed with paraquat diet (0.25 g/kg diet) and with control diet for 11 days. TBARS, thiobarbituric acid reactive substances. Boxplot widths are proportional to the square root of the samples sizes. Whiskers indicate the range outside 1.5 times the inter-quartile range above the upper and below the lower quartile. Outliers were plotted as dots.

Biology Open • Supplementary information
